# From FINGER to World-Wide FINGERS - multidomain interventions for risk reduction and prevention of dementia

**DOI:** 10.1192/j.eurpsy.2026.10995

**Published:** 2026-07-31

**Authors:** I. M. Marques

**Affiliations:** Department of Psychiatry, Unidade Local de Saúde de Coimbra, Coimbra, Portugal

## Abstract

**Introduction:**

Dementia affects more than 50 million individuals worldwide. It is estimated that almost half of the cases can be attributed to potentially modifiable factors, highlighting the importance of targeting these factors in dementia prevention.

**Objectives:**

The aim is to provide a review of multidomain interventions for risk reduction and prevention of dementia.

**Methods:**

Literature review on the subject, using databases such as PubMed.

**Results:**

At present, there is no cure for dementia. Still, making healthy lifestyle choices is crucial for delaying or preventing its onset. In this context, the Finnish Geriatric Intervention Study to Prevent Cognitive Impairment and Disability (FINGER) was launched in Finland, lasting two years and including individuals at high risk of developing dementia, identified through the Cardiovascular Risk Factors, Aging, and Dementia (CAIDE) risk score (Kivipelto M, Mangialasche F, Ngandu T. Lancet Neurol 2018; 17:27). The study focused on regular physical exercise, a Mediterranean-style diet, cognitive and social stimulation and the management of cardiovascular risk factors as measures to prevent or reduce the risk of dementia. The results were promising, showing beneficial effects on cognitive performance, as even small individual changes across multiple risk factors can lead to favorable outcomes when combined (Kivipelto M, Mangialasche F, Ngandu T. Lancet Neurol 2018; 17:27). Since the FINGER study demonstrated that prevention is possible through multidomain interventions, it became essential to adapt, expand, and optimize these findings through the World-Wide FINGERS initiative (Kivipelto M et al. Alzheimers Dement 2020; 16:1078-94). This project incorporates diverse cultural, geographical, and economic settings, providing a platform to identify individuals with different risk profiles and to encourage countries to implement multidomain lifestyle interventions. It also facilitates data and knowledge sharing by identifying globally applicable strategies to prevent or delay the onset and progression of late-life cognitive impairment.

**Conclusions:**

Dementia represents a major public health challenge. The risk of dementia begins to take shape long before old age, highlighting the importance of early prevention. Identifying individuals at higher risk may allow for the development of strategies to prevent or delay the onset of dementia. However, it remains essential to include more low- and middle-income countries, adapting lifestyle interventions to different contexts and ensuring personalization. Longer follow-up periods are also needed to gather further results. Digital platforms could also become a valuable resource for improved monitoring.

**Disclosure of Interest:**

None Declared

